# Antidepressant Active Ingredients From Chinese Traditional Herb *Panax Notoginseng*: A Pharmacological Mechanism Review

**DOI:** 10.3389/fphar.2022.922337

**Published:** 2022-06-16

**Authors:** Yanwei Li, Qingwan Guo, Junqing Huang, Ziying Wang

**Affiliations:** ^1^ Guangzhou Key Laboratory of Formula-pattern Research Center, School of Traditional Chinese Medicine, Jinan University, Guangzhou, China; ^2^ Interdisciplinary Institute for Personalized Medicine, School of Traditional Chinese Medicine, Jinan University, Guangzhou, China

**Keywords:** depression, chinese herbal medicine, Panax notoginseng, active ingredients, antidepressant

## Abstract

Depression is one of the most common mental illnesses in the world and is highly disabling, lethal, and seriously endangers social stability. The side effects of clinical drugs used to treat depression are obvious, and the onset time is longer. Therefore, there is a great demand for antidepressant drugs with better curative effects, fewer side effects, and shorter onset time. *Panax notoginseng*, a Chinese herbal medication, has been used to treat depression for thousands of years and shown to have a therapeutic effect on depression. This review surveyed PubMed’s most recent 20 years of research on *Panax notoginseng*’s use for treating depression. We mainly highlight animal model research and outlined the pathways influenced by medicines. We provide a narrative review of recent empirical evidence of the anti-depressive effects of *Panax Notoginseng* and novel ideas for developing innovative clinical antidepressants with fewer side effects.

## 1 Introduction

Depression is one of the most prevalent psychiatric disorders globally (Parker and Paterson, 2015). Patients suffering from depression always exhibit unhappiness, low self-esteem, and high self-criticism, and severe cases may even exhibit suicidal impulses, causing social instability (Limon et al., 2016). The fundamental causes of depression are still unknown. In contrast, much research in recent decades has indicated that various factors such as biological, psychological, and social environment may lead to depressive disorders (Wang Q. et al., 2017). Many theories have been proposed to explain the pathophysiology of depression, including chronic inflammation, immune system abnormalities, and a lack of neurotransmitters such as serotonin (5-HT) (Allison and Ditor, 2014; Jeon and Kim, 2016). Studies showed that long-term exposure to elevated inflammatory cytokines could lead to depression (Felger and Lotrich, 2013).

Selective serotonin reuptake inhibitors (SSRIs), monoamine oxidase inhibitors (MAOI), and tricyclic antidepressants (TCA) are the most commonly used clinical treatments for depression, but they all have different adverse effects (Jakobsen et al., 2020). For example, TCA causes adverse symptoms such as dry mouth, dizziness, blurred vision, constipation, and drowsiness. Hypotension, weight gain, and sexual dysfunction are among the side effects of MAOIs. Although SSRIs do not have various harmful effects, excessive use of these drugs would lead to drug resistance (Feighner, 1999). Existing antidepressant medications typically have drawbacks, such as prolonged onset and adverse effects. It is still critical to discover new curative drugs with fewer adverse effects.

Chinese herbal medicine (CHM) has a long history of nontoxic use in treating depression-like syndromes (Singh and Zhao, 2017). Treatment through CHM has advantages, such as fewer adverse effects and multiple therapeutic effects (Pang et al., 2015). *Panax notoginseng (Burk.) F. H. Chen*, also called ***Sanqi***, is a comparatively expensive Chinese herbal medicine used for an extended period blindly (Wang T. et al., 2016). *Panax notoginseng* has medicinal properties such as eliminating blood stasis, reducing bleeding, and alleviating pain (Lelu et al., 2016). Modern research showed that the active ingredients in *Panax notoginseng* were primarily *Panax notoginseng* saponins (PNS) (Qu et al., 2020). There are more than 100 distinct saponins in PNS, the most abundant of which are ginsenoside Rb1, ginsenoside Rg1, notoginsenoside R1, ginsenoside Rd, and ginsenoside Re (Qu et al., 2020). These saponins offer a variety of beneficial effects, including antioxidant and anti-inflammatory properties, in addition to their benefit to the central nervous system, cardiovascular system, and immune system (Zhou et al., 2019).

Many investigations revealed that PNS evolved to treat insomnia, anxiety, and neurasthenia neurosis (Zhang et al., 2018). PNS has been identified to enhance neuronal plasticity and promote Nestin’s expression and brain-derived neurotrophic factor (BDNF). Furthermore, it has been proposed that PNS enhanced monoamine neurotransmitters such as 5-hydroxytryptamine (5-HT), norepinephrine (NE), dopamine, and exerted antidepressant effects. Therefore, the anti-depression effect of PNS is worthwhile to investigate further and serves as a model for future research. This review summarized and analyzed the literature on *Panax notoginseng* and its active compounds. The following paragraph presents their pharmacology and therapeutic effect in animal and human studies (Ruan et al., 2019; Zhang B. et al., 2021; Xia et al., 2021).

## 2 Animal Model of Depression

When studying depression, animal models provide an integrated approach to examine molecular and cellular mechanisms of depressive disorder. The handling of pharmacological drugs and gene editing is practically impossible to apply in the human patient, which can be operated in animal models. Considering the etiology of depression, rodent models have been created based on acute or chronic stress exposure, gene-environmental connection, exogenous administration of glucocorticoids, and genetic modifications.

**FIGURE 1:**
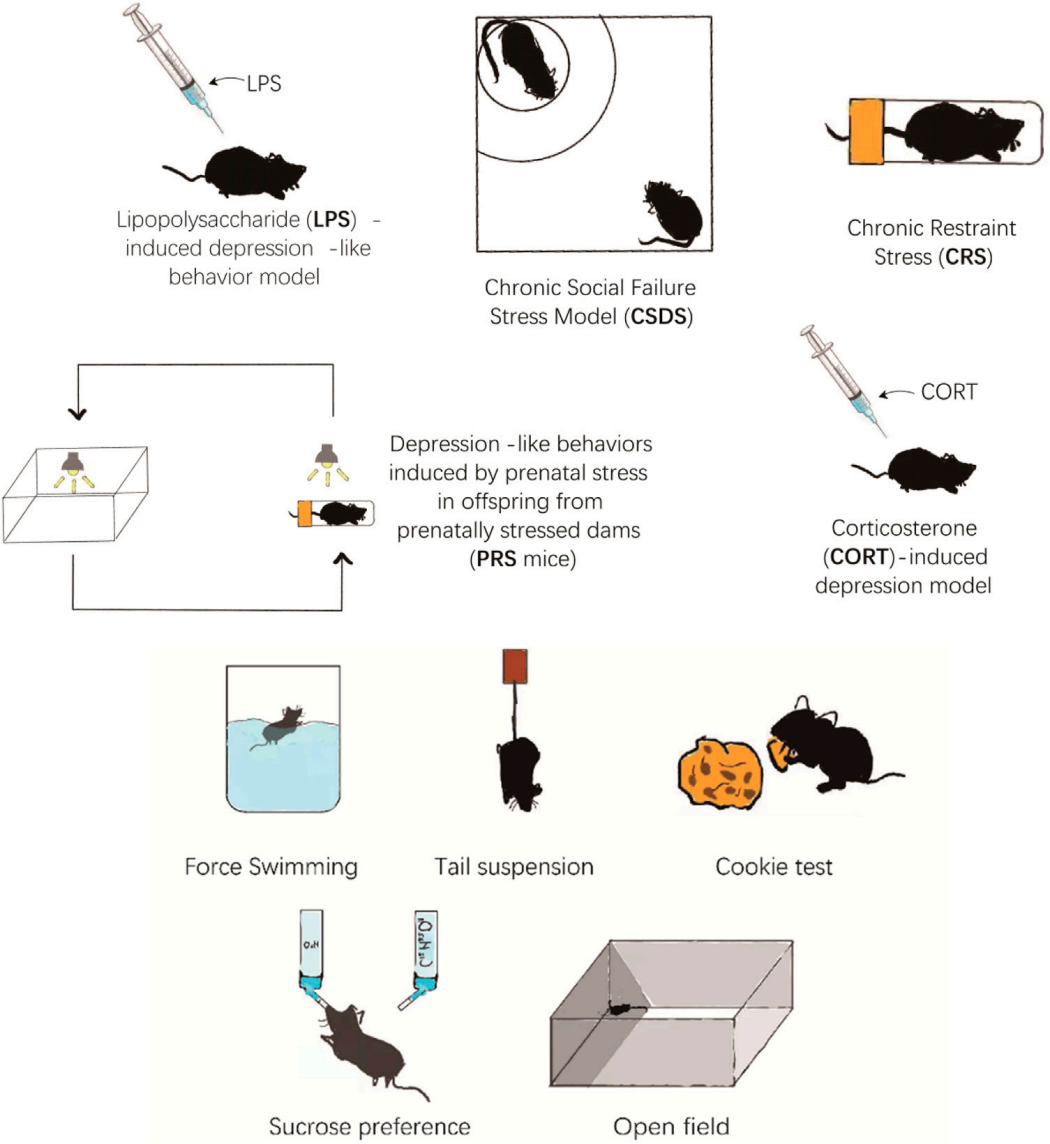
The different animal model of depression.

The most commonly used depressive animal models are chronic unpredictable mild stress (CUMS) model, chronic social failure stress Model (CSDS), chronic restraint stress (CRS), depression-like behaviors induced by prenatal stress in offspring from prenatally stressed dams (PRS), lipopolysaccharide (LPS)-induced depression-like behavior model, and corticosterone (CORT)-induced depression model (Figure 1). Each model has its own set of advantages and disadvantages. Using appropriate behavioral tests for experiments may result in more reliable and solid pre-clinical reference data.

### 2.1 Chronic Unpredictable Mild Stress (CUMS) Model

**FIGURE 2:**
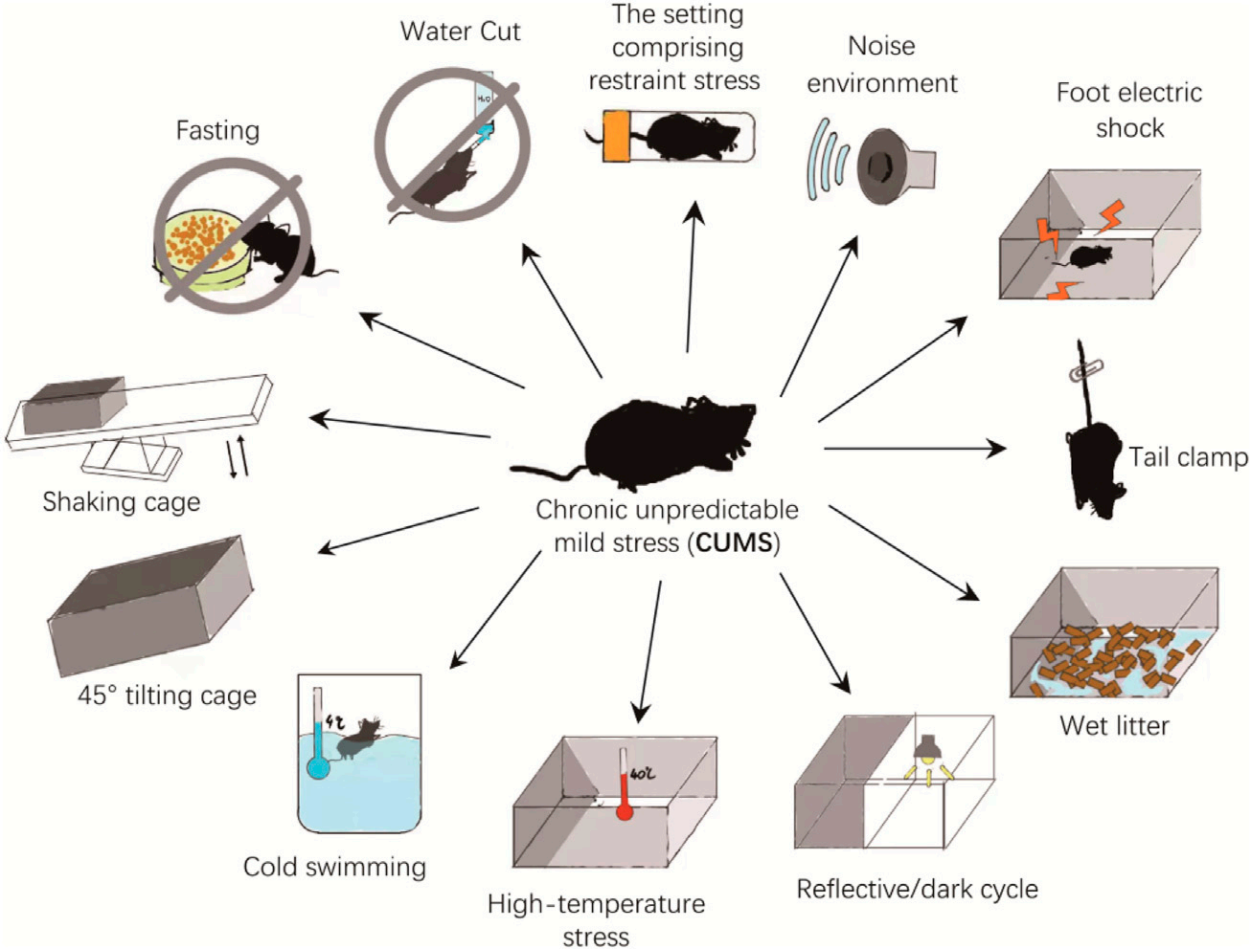
The CUMS animal model.

The CUMS is one of the most widely used models in depression studies. The procedure is to place each experimental and model group separately and repeatedly exposed to a set of CUMS: the setting comprising restraint stress (4 h), noise environment (110dB, 1 h), foot electric shock (3mA, one electric shock/5 s), tail clamp (clamp the tail 1 cm from the tip of the tail for 3 min), wet litter (24 h), reflective/dark cycle (24 h), high-temperature stress (40°C, 20 min), cold swimming (4°C, 5 min), 45° tilting cage (12 h), shaking cage (15 min), fasting (24 h), and water cut (24 h). The mice are randomly stimulated by two pressure sources every day, and the entire modeling process lasts for 5 weeks (Figure 2). The experimental groups receive medicine during the modeling period, whereas control and model groups receive normal saline (Antoniuk et al., 2019). However, studies have shown that water cut and fasting can affect ghrelin levels, potentially affecting results. Whether to eliminate these two stressors during modeling deserves further study ((Huang et al., 2019).

### 2.2 Lipopolysaccharide (LPS)-Induced Depression-like Behavior Model

Lipopolysaccharide (LPS) is a cytokine inducer (Arioz et al., 2019). Numerous studies have revealed that LPS can induce reliable depression-like behaviors at various periods and dosages. Mice are administered LPS, dissolved in nontoxic saline through Intraperitoneal injections. The daily injection time of the drug is fixed at nine or 10 a.m. (Tang et al., 2020). Exposure to LPS induces a decrease in sucrose preference and a reduction in sensitivity to rewarding brain stimulation (Limon et al., 2016).

### 2.3 Chronic Social Failure Stress Model (CSDS)

CSDS is another mouse model of depression used to induce depression-like behaviors, such as social avoidance and anhedonia (Wang H. et al., 2021). The intruder (the experimental mouse) can interact with another aggressive and large CD1 mouse for 10 min during each defeat phase. The invader is rapidly attacked and defeated by the resident CD1 mice during this time. The experimental mice are exposed to various resident attackers for 10 min each day. The entire modeling process takes 10 days. Subsequently, mice exhibit depression-like behavior, referred to as “susceptibility" (Wang Q. et al., 2017).

### 2.4 Chronic Restraint Stress (CRS)

CRS is a depressive mice model to examine behavior, brain morphology, and hormone alterations (Deng et al., 2021). Mice are usually confined in the device (a cylindrical, transparent, acrylic tank (height = 8.5 cm, diameter = 2.5 cm) fixed on a square pedestal) for 1–6 h. This process lasts for 14–21 days or even an extended period. Mice exposure to CRS can induce depression-like behaviors, such as anhedonia (Jeon and Kim, 2016).

### 2.5 Depression-Like Behaviors Induced by Prenatal Stress in Offspring From Prenatally Stressed Dams (PRS Mice)

The offspring of the prenatal stress dam will display depression-like behaviors in adulthood. This model is known as PRS mice (Dong and Pandey, 2021). The pregnant mice are grown individually and can eat and drink freely. The mice in the control group are not disturbed during pregnancy and have a 12-h light-dark cycle. The model and experimental group mice are placed in separate rooms with fluorescent ceiling lights. The entire model-building process occurs from the fifth day of pregnancy to after delivery. The mice are confined in a transparent tube. Each 30-min lesson is scheduled three times daily. During pregnancy, the light in the mouse room is constant. After the offspring of mice are weaned, male mice are selected for the study. The principle of random allocation is adopted, with four to five mice per cage. After 40 days of birth, the mice can start the experiment (Allison and Ditor, 2014).

### 2.6 Corticosterone (CORT)-Induced Depression Model

The model-building procedure randomly divides mice into three groups: model group, experimental group, and control group. The mice are injected subcutaneously with CORT once a day in the model and experimental groups. After 4 weeks, CORT model is accomplished. The mice in the control group are injected with saline, and the other conditions remain the same. Mice injected with CORT exhibit depression-like behavior (Lee et al., 2020). In addition to subcutaneous injection, oral CORT is also a commonly used route of administration and can also produce depression-like behaviors ((Weina et al., 2018), (Mei et al., 2014)).

### 2.7 Chronic Constriction Injury (CCI) Model

The construction of this model requires 7–8 week old mice. Mice were anesthetized by intraperitoneal injection of sodium pentobarbital. The right sciatic nerve of the mice in the experimental group was exposed in the middle of the thigh, and three ligations were given near the trigeminal nerve until a brief twitch was observed in the corresponding area. The surgical site was treated with streptomycin and the wound was sutured. In the control group, the sciatic nerve was exposed but not ligated, and the rest of the operations were the same. Ligated mice are more likely to exhibit depression-like behavior while developing chronic contractile damage (Zhao et al., 2014a). It was important to note that all surgical procedures should be performed by the same person (Zhao et al., 2014b)

### 2.8 The L-Alpha-Aminoadipic Acid (L-AAA)–Infused Mice Model

This model is characterized by a reduction in astrocytes in the brains of mice injected with L-AAA, resulting in depression-like behaviors. Eight-week-old male C57Bl/6 mice randomly divided into experimental group and control group. The mice in the experimental group were anesthetized with intra-peritoneal by intraperitoneal injection. The cannula was then implanted into the prefrontal cortex (PFC) of the mice using a stereotaxic apparatus (David et al., 2019). Seven days after the mice recovered, L-AAA was injected into the PFC once a day for 2 days using an injection cannula and a microdrive pump. The control group was treated with a sham operation and implanted with a cannula, but no injection treatment was performed, and the rest of the operations were the same (Kim et al., 2020).

### 2.9 The Learned Helplessness (LH) Model

In this model, male rats were used, and rats exposed to the LH paradigm increased microglia in the hippocampus. An increase in microglia in the hippocampus is associated with altered stress, and its increase produces an inflammatory response that further develops into depression (Iwata et al., 2016). The construction of the LH model requires the use of the Gemini avoidance system. The system is divided into two compartments with an openable door in the middle. During the first 2 days, rats received 60 unavoidable foot shocks (0.65 mA, duration 20–40 s, average: 30 s). On the third day, a two-way conditioned avoidance test was performed, which consisted of 30 experiments. Rats that failed to escape more than 20 times in 30 experiments were considered to have acquired the LH state, that is, the model was successfully established (Iwata et al., 2016).

### 2.10 Prolonged Social Isolation in Adult Rodents Model

For this model, 7–8 week old Sprague-Dawley rats were used and kept in cages with alternating light and dark. Rats were randomly divided into an experimental group and a control group. Only one rat in the experimental group was housed in each cage. The rats in the control group were housed two per cage. Relevant behavioral tests were performed after 10–12 weeks of rearing (Mao and Wang, 2018). Rats in this model will have the coexistence of anxiety-like behaviors and depression-like behaviors, but the mechanisms are different (Wallace et al., 2009).

### 2.11 Trauma Witness Model (TWM)

TWM is modified from the social failure model (Patki et al., 2015). Larger LE male rats are typically used as residents and smaller Sprague-Dawley male rats as intruders. The specific process of the experimental group was to raise two Sprague-Dawley rats in the same cage to establish contact. One of the Sprague-Dawley rats was then treated as an intruder and brought into the cage of the LE rats, resulting in social defeat behavior. After the failure, the intruders and residents were separated by baffles. Another Sprague-Dawley rat was placed in the enclosure around the resident’s cage as a trauma witness to witness the social failure of the intruder. The intruder and the witness were then put into the same cage. The above was a trauma witnessing process. The whole modeling process lasted for 7 days, and a trauma witness was performed every other day, for a total of 3 times. A control group did not undergo social defeat but was placed in a resident cage. The difference between the control group and the experimental group was that the residents were taken out, and the rest of the operations were the same as in the experimental group (Patki et al., 2014).

## 3 Antidepressant Active Ingredients From *Panax Notoginseng*

### 3.1 Total Saponins From the Caudexes and Leaves of *Panax notoginseng* (SLPN)

Ginsenosides are the primary biologically active dammarane triterpenoids extracted from *Panax notoginseng*. It is a biologically active component in the roots of *Panax notoginseng*. Several studies have proven the therapeutic effects of ginsenosides on many central nervous diseases, including mental diseases, especially major depression (Kim and Cho, 2021; Terstege et al., 2021). However, its underlying mechanism remains unclear. (Xiang et al., 2011; Zhang et al., 2018). Dammarane triterpenoids include 20(S)-protopanaxatriol saponins, such as ginsenosides Rh1, Rg1, and Re, and 20(S)-protopanaxadiol saponins, such as notoginsenoside R1, ginsenosides Rb1, and Rg1 (Wei et al., 2018a; Wei et al., 2018b; Zhang et al., 2018; Cao et al., 2019; Ren et al., 2020).

Experiments have revealed that SLPN has antidepressant effects in various rodent depression models. Its effect may be achieved by boosting brain monoamine neurotransmitters serotonin, dopamine, and norepinephrine (Xiang et al., 2011). In CUMS mice, SLPN enhanced the protein levels of CREB1 and BDNF by increasing the expression of down-regulated circRNA (mmu_circ_0001223) in CUMS mice and exerted an antidepressant effect (Zhang et al., 2018). Recent research has revealed that PNS may modulate intracellular Ca^2+^ concentrations and act as an antidepressant (Xiang et al., 2011).

In CUMS model, ginsenosides modulate the excitability of amino acid and monoamine neurotransmitter metabolites, significantly lowering the alterations in brain and peripheral metabolites caused by stress (Wang et al., 2012). In another study, ginsenosides not only enhanced the concentration of monoamine neurotransmitters in the hippocampus of CMS mice but reversed the expression of BDNF(Dang et al., 2009). In addition, ginsenosides significantly reduced the depression-like behavior of LPS model mice. After LPS model mice were treated with ginsenosides, pro-inflammatory cytokines were reduced. The study displayed that the antidepressant effect of ginsenosides might be linked to peripheral anti-inflammatory (Kang et al., 2011).

### 3.2 Single Compounds

**FIGURE 3:**
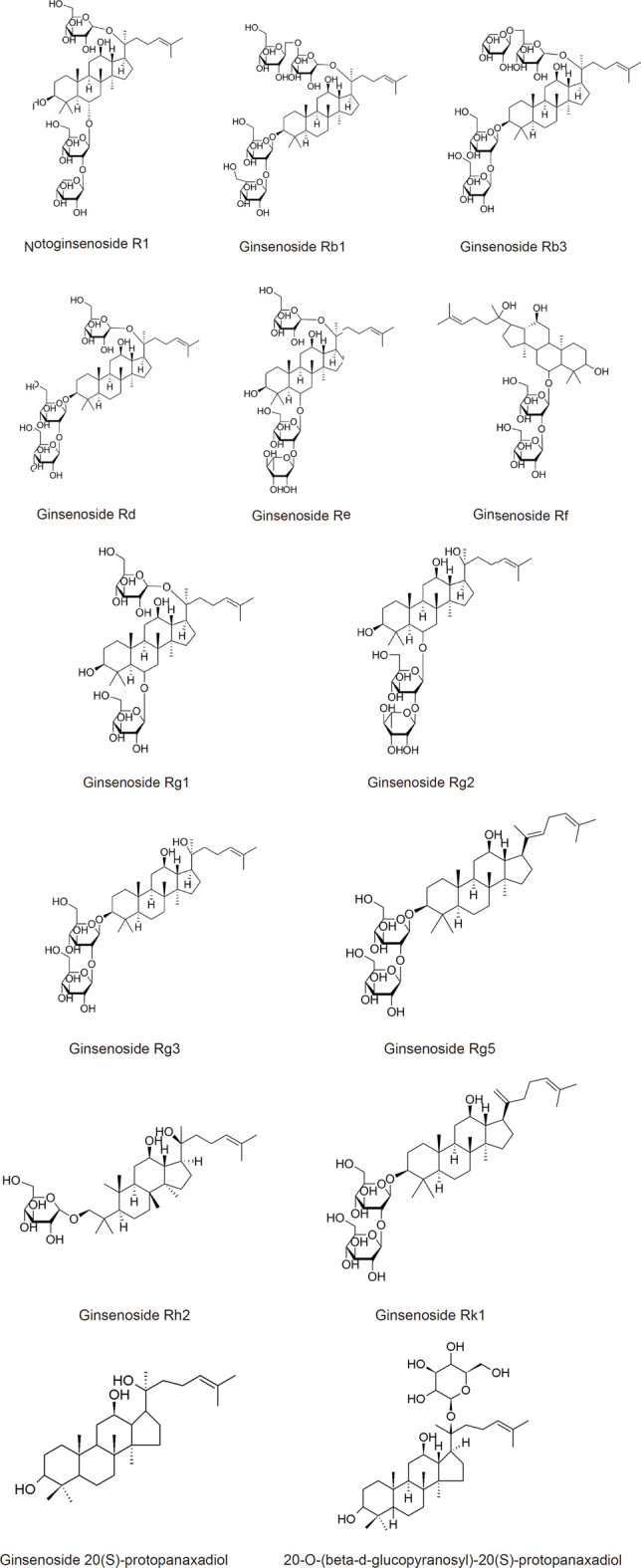
(Continued).

**FIGURE 4:**
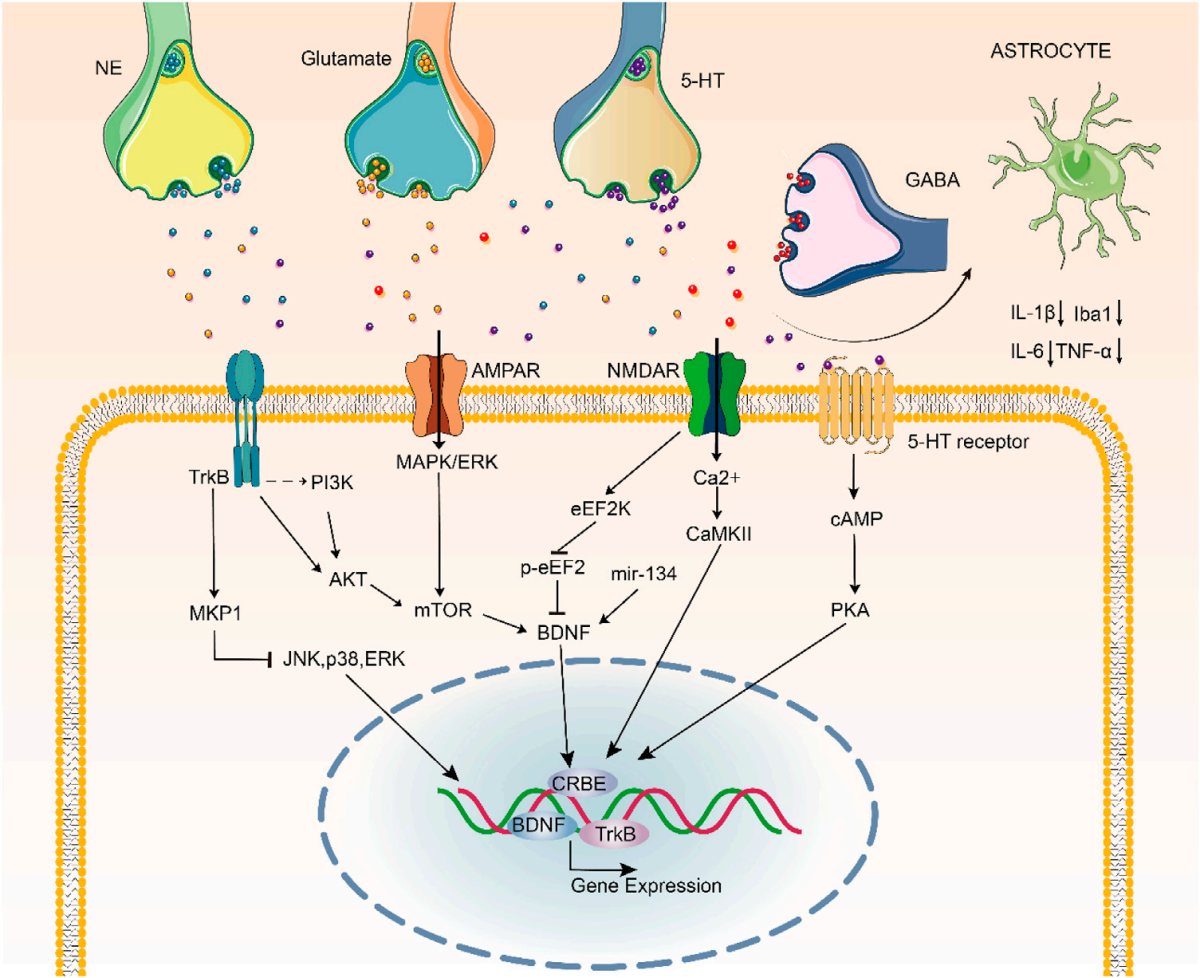
Signaling pathways underlying the pathophysiology and treatment of depression.

Exclusive of admixtures, we also summarized recent 20 years’ PubMed research focus on the single active ingredients extracted or metabolized from *Panax notoginseng*. We have concluded these single compound’s structures and their main mechanisms are listed in Table 1 and illustrated in Figure 3 and Figure 4.

**TABLE 1:** The illustration of molecular mechanisms and outcomes of activated *Panax notoginseng* ingredient.

Original Sources	Compounds	Models	Molecular Mechanisms and Outcomes	Dose and Duration	Refs
*Panax notoginseng*	Panax notoginseng saponins	CMS rats	5-HT, DA, and NE ↑	10,30,100,300,1000 mg/kg	Xiang et al. (2011)
			mmu_circ_0001223, CREB1 and BDNF ↑	30 mg/kg 3 weeks	Zhang et al. (2018)
	Notoginsenoside R1	db/db mice	Akt/Nrf2 ↑	10 and 30 mg/kg 10weeks	Zhai et al. (2018)
			NLRP3 ↓		
	Ginsenoside	CUMS rat	5-HT,5-HIAA,NE, DA ↑	100 mg/kg 27days	Wang et al. (2012)
	Ginsenoside Rb1	CUMS rat	Amino acids and monoamine neurotransmitter metabolites ↓	4, 8, 16 mg/kg, p.o	Wang G. L. et al. (2017)
			5-HT, 5-HIAA, NE, DA, and GABA ↑	5,10,20 mg/kg	Wang et al. (2018)
			Glu ↓		
		CRS rat	BDNF, p-AKT/AKT ↑	10 mg/kg 14 days	Guo et al. (2021)
			IL-1β, TNF-α, ionized calcium-binding adapter molecule 1 ↓		
	Berberine and ginsenoside Rb1	Rat diabetes mode	BDNF ↑		Zhang J. H. et al. (2021)
			Plasma Cortisol and ACTH ↓		
	Ginsenoside Rb3	CUMS mice	5-HT, DA, BDNF ↑		Cui et al. (2012)
			The basal synaptic transmission ↓	10,30,50 μM	Jiang et al. (2012)
			Serum ACTH and corticosterone, NA ↑	50,100 mg/kg p.o	Zhang et al. (2016)
*Panax notoginseng*	Ginsenoside Rd	IS, EC rat	BDNF, BDNF^+^/NeuN^+^ cell population ↑	5 mg/kg p.o	Han et al. (2020)
			CORT, NF-κB activation, NF-κB^+^/CD11c^+^ cell population ↓		
	Ginsenoside Re	IS rat	BDNF↑, TH ↓	10, 20, 50 mg/kg 10 days	Lee et al. (2012)
	Ginsenoside Rf	L-AAA rat	GFAP,Ki-67, astrocyte changes in the hippocampus ↓	20 mg/kg p.o	Kim et al. (2020)
		CCI rat	IL-10 ↓	0.5, 1.5, 3 mg/kg	Li et al. (2019)
	Ginsenoside Rg1	LPS, SPS Rat	IL-1β and TNF-α, astrocytes, microglia, PSD95, Arc, GluA1, Kir4.1, GluN2A ↓	10,20, 40 mg/kg/d i.p. for 14 days	Zhang Z. et al. (2021)
		CSDS rat	p-ERK1/2 ↑	20, 40 mg/kg i.g	Jiang et al. (2020a)
			IL-6, TNF-α, IL-1β, iNOS, COX2, caspase-9 and -3, lba1, p-JNK1/2, p-P38 MAPK, NF-κB ↓		
		CUMS rat	pro-inflammatory cytokines, microglia and astrocytes↓	40 mg/kg	Fan et al. (2018a)
			Bcl-2, Nrf2 ↑		
			Neuronal apoptosis, Caspase-3, Caspase-9, (p-p38 MAPK),κB (NF-κB) p65 ↓		
			IL-1β, NF-κB ↓		Zhang et al. (2019)
			BDNF ↑	40 mg/kg i.p. 5 weeks	Liu et al. (2016)
			mir-134 ↓	40 mg/kg 5 weeks	Fan et al. (2018b)
			GR ↑	5,10,20 mg/kg p.o	Mou et al. (2017)
*Panax notoginseng*		GDX rat	AR ↑		
		CUMS, GDX rat	Serum testosterone ↑		
			Serum CORT↓		
		CMS rat	BDNF ↑	2.5, 5, 10, 20 mg/kg i.p	Jiang et al. (2012)
			CORT ↓		
		CORT	Cx43 ↓	0.1, 1, 10 μM	Xia et al. (2020)
			Cx43 ↓	0.1, 1, 10 μM	Wang H. Q. et al. (2021)
			the gap junction dysfunction ↓	0.1, 1, 10 μM	Xia et al. (2017)
		CORT,CUS	primary astrocytes Cx43 ↓	20 mg/kg/day	Lou et al. (2020)
		CUS	Cx43 ↓	5,10,20 mg/kg 28days	Jin et al. (2017)
	Ginsenoside Rg2	CMS rat	BDNF ↑	10, 20 mg/kg	Ren et al. (2017)
	Ginsenoside Rg3	LPS rat	IL-6, TNF-α, Pro-inflammatory cytokines ↓	20, 40 mg/kg i.g	Kang et al. (2017)
		CMS rat	NMDA the cell cycle ↓	50,100, 150 mg/kg	Zhang et al. (2017)
			BDNF, p-CREB ↑		
		CSDS rat	BDNF ↑	10, 20 mg/kg i.p	You et al. (2017)
*Panax notoginseng*			NA ↑	50,100 mg/kg	Zhang et al. (2016)
			ATCH, CORT ↓		
	Ginsenoside Rg5	CSDS rat	BDNF ↑	5,10,20,40 mg/kg	Xu et al. (2017)
	Ginsenoside Rh2	CRC rat	IL-6,IL-1,TNF-α ↓	0.2,1,5 mg/kg	Wang J. et al. (2016)
	Ginsenoside Rk1	LPS rat	SOD,BDNF, TrkB ↑	5,10,20 mg/kg	Li Z. et al. (2020)
			TNF-α, IL-1, IL-6, Sirt1, p-NF-κb/NF-κb, ↓		
			p-IκB-α/IκB-α ↓		
	Ginsenoside 20(S)-protopanaxadiol	CUMS rat	Sirt1 ↑	20,40 μmol/kg	Jiang et al. (2020a)
			CORT,IL-6,IL-1β, TNF-α,5-HT, NE,Iba1, iNOS, COX2, cleaved-caspase3, cleaved-caspase9, ↓		
			Bax,Bcl-2, ac-p65↓		
		rat olfactory bulbectomy	CORT ↓		Xu et al. (2010)
		depression model			
	Ginsenoside Metabolite Compound K	CUMS rat	5-HT,DA, GSH,GPx, BDNF, NGF ↑	10, 30 mg/kg	Song et al. (2018)
			MAO B ↓		
			NA ↑	50, 100 mg/kg	Zhang et al. (2016)
	Ginseng extract G115^®^		enhance the effect of fluoxetine		Terstege et al. (2021)
	Ginsenoside H dripping pills (GH)		DA,NE,5-HT ↑	28,56,112 mg/kg	Zhao et al. (2020)

#### 3.2.1 Notoginsenoside R1

Notoginsenoside R1 (Sanchinoside R1) is a novel ingredient with cardiovascular activity in *Panax notoginseng*. Notoginsenoside R1 was reported to exert numerous biological effects such as favorable alterations in neuronal plasticity and display neuroprotective effects in brain injuries (Liu et al., 2020). Recent research chiefly examined the function of an antidepressant in depressive mice models. The results showed that Notoginsenoside R1 exhibits a neuroprotective effect and enhanced depressive-like behavior through activating Akt/Nrf2 signaling pathway and inhibiting the activation of NLRP3 inflammasomes in db/db mice (Zhai et al., 2018). Subsequently, Zhan et al. found that NR1 can mediate the symptoms of depressive behaviors in CUMS mice model primarily through PI3K/AKT/NF-kB pathway (Zhan et al., 2021).

#### 3.2.2 Ginsenoside Rb1

Ginsenoside Rb1 (Rb1) is the most abundant and activated monomer in *Panax Notoginseng* with a demonstrable biological activity against depression. Experimental results based on a CUMS rat model demonstrate that Rb1 increases the levels of 5-HT, 5-HIAA, NE, DA, and GABA in the rat brain and reduces the expression of Glu, exhibiting an antidepressant-like effect. Simultaneously, homovanillic acid (HVA) and 3,4-Dihydroxyphenylacetic acid (DOPAC) did not express any abnormalities (Wang G. L. et al., 2017; Wang et al., 2018). Additionally, a diabetic rat model combined with CUMS modeling will reduce plasma cortisol and adrenocorticotropic hormone (ACTH) levels. Berberine treatment with ginsenoside Rb1 (B + GRb1) could increase BDNF expression, improve glucose metabolism, insulin resistance and reduce depressive-like behavior (Zhang J. H. et al., 2021).

Furthermore, it was reported that Rb1 effectively amplifies the protein expression of BDNF and p-AKT levels in CRS mice model while also inhibiting LPS-induced IL-1β and TNFα in BV2 cells and mice serum. Finally, studies show that Rb1 can prevent CRS-induced depression in mice because it inhibits the protein expression of IL-1β and TNF-α in BV-2 microglia induced by LPS (Guo et al., 2021).

#### 3.2.3 Ginsenoside Rb3

Ginsenoside Rb3 is the most abundant component of *Panax notoginseng*, accounting for more than 15% of its total content (Dang et al., 2009; Cui et al., 2012). It is another potential antidepressant candidate (Zhang et al., 2016). Like Rb1, Rb3 has been identified to have antidepressant-like effects (Cui et al., 2012; Zhang et al., 2016). In the model induced by the reserpine and chronic mild stress model, Rb3 elevated the expression of BDNF and other monoamine neurotransmitters serotonin, dopamine, and norepinephrine.

The whole-cell route clamp recording experiment further investigated that Rb3 can be perfused to stimulate the action potential transmitter of neurons in the somatosensory cortex. However, Rb3 inhibits basal synaptic transmission, encourages PS recovery after OGD in a concentration-dependent manner, and causes PS depression.

In a whole-cell patch-clamp recording, it was found that the action potential conduction of neurons in the somatosensory cortex was occupied and blocked by Rb3. The experiment indicated the mechanism of Rb3 in antidepressant-like effects (Cui et al., 2012). In addition, results have demonstrated that Rg3 can significantly boost NA levels but do not affect 5-HT and DA levels in the mouse brain (Zhang et al., 2016).

#### 3.2.4 Ginsenoside Rd

Ginsenoside Rd is a protopanaxadiol abundantly found in various Panax species, particularly *Panax Notoginseng*. It has diverse pharmacological effects, including neuroprotective and anti-tumor effects. Studies have revealed that in immobilization and Escherichia coli (EC) induced anxiety or depression mice, Rd could reduce depression and colitis by regulating NF-kB-mediated BDNF expression and intestinal Flora imbalance, eventually ameliorating depressive behaviors (Han et al., 2020).

#### 3.2.5 Ginsenoside Re

Ginsenoside Re belongs to 20(s)-protopanaxatriols group, which is significant ginsenoside. According to literature, Re has various pharmacological actions that occur through several pathways. In the immobilization stress (IS) rat model, Re administration might recover the expression of tyrosine hydroxylase (TH) in locus coeruleus (LC) and boost BDNF expression in the hippocampus. Re pretreatment significantly improved helpless behavior and cognitive impairment by regulating the central noradrenergic system (Lee et al., 2012).

#### 3.2.6 Ginsenoside Rf

Ginsenoside Rf is a steroid glycoside found in plants of the genus Panax exhibiting various biological activities. Recently, Rf has been used to treat neuropsychiatric disorders. Studies have indicated that in mouse models injected with L-AAA, Rf can increase the expression of the glial fibrillary acidic protein (GFAP) and nuclear protein Ki-67 in the prefrontal cortex (PFC), which L-AAA reduces. Concurrently, Rf improved the density of astrocytes in the hippocampus and decreased depression-like behavior (Kim et al., 2020).

Furthermore, in a rat model of chronic constriction injury (CCI) of the sciatic nerve, Rf could inhibit the growth of pro-inflammatory cytokines in the spinal cord and DRG and increase IL-10 in DRG. Mechanical allodynia could also be efficiently relieved while reducing depression-like behavior (Li et al., 2019).

#### 3.2.7 Ginsenoside Rg1

Ginsenoside Rg1 is one of the most significant active ingredients of the genus Panax (Xia et al., 2017; Zhang et al., 2019; Jiang et al., 2020b). It has numerous protective effects on neurological diseases, including anti-depression effects ((Jiang et al., 2012; Liu et al., 2016; Xia et al., 2017; Fan et al., 2018a; Fan et al., 2018b; Zhang et al., 2019; Li Y. et al., 2020; Jiang et al., 2020b). Rg1 is the primary compound that has been extensively studied in different depression models.

Although Rg1 exhibits little toxicity, there are no evident adverse effects that effectively prevent depression. In LPS and SPS depression models, Rg1 could fade fear and alleviate depression-like behaviors. The protective mechanism primarily reduces the expression of IL-1β and TNF-α induced by LPS or SPS stimulation. Besides, Rg1 mediated the activation of astrocytes and microglia and elevated the expression of hippocampal synaptic proteins (such as PSD95, Arc, and GluA1). Rg1 could also inhibit the expression of Kir4.1 and GluN2A in the hippocampus. In particular, the reduction of Kir4.1 efficiently alleviated the fear of LPS mice and improved their depression-like behavior. Injection of TNF-α into the brain ventricle of mice improved the expression of Kir4.1 in the hippocampus. Therefore, it is concluded that Rg1 may reduce Kir4.1 and TNF-α in the hippocampus by encouraging synaptic proteins and serving as an antidepressant (Zhang Z. et al., 2021).

Furthermore, in CSDS model, Rg1 inhibited pro-inflammation cytokine IL-6, TNF-α, and IL-1β, and reduced the protein expression of iNOS, COX2, caspase-9, and caspase-3. Moreover, it regulated the activation of microglia in the hippocampus (Iba1), downregulated p-JNK1/2 and p-P38 MAPK protein expression, increased p-ERK1/2 level, and NF-kB in the hippocampus. Thus, Rg1 protected mice from depression when they were exposed to CSDS. Rg1 could also regulate SIRT1 and reduce the level of acetylated p65 (ac-p65) in the hippocampus (Jiang et al., 2020b).

Besides the depressive models mentioned above, current research has revealed that Rg1 has an antidepressant impact on depressive mice generated by CUMS (Liu et al., 2016; Fan et al., 2018b). Chronic pretreatment with Rg1 before CUMS could effectively inhibit the activity of inflammatory pathways by reducing the overexpression of pro-inflammatory cytokines and the activation of microglia and astrocytes. Rg1 treatment also boosted the synapse-related protein in the ventral medial prefrontal cortex (vmPFC) (Fan et al., 2018a). In CUMS model, Rg1 can inhibit neuronal apoptosis induced by CUMS exposure, essentially by increasing Bcl-2 and Nrf2, whereas reducing the cleaved caspase-3, caspase-9, p-p38 MAPK, and NF-κB p65 subunit (Fan et al., 2018a). Rg1 can also reverse CUMS-induced IL-1β increase; the mechanism could involve suppressing nuclear factor kappa B pathway activation and regulating nucleotide-binding and oligomerization domain-like receptor family pyrin domain-containing three inflammasome expressions (Zhang et al., 2019). In CUMS rat model and the gonadectomized mouse model, Rg1 considerably improved their depression-like behavior, and both reduced the serum corticosterone level. Though, in CUMS model, Rg1 also raised the secretion of serum testosterone and increased glucocorticoid receptor (GR) protein levels in PFC and hippocampus in rats. In GDX model, Rg1 did not affect serum testosterone, but mice increased the androgen receptor (AR) of PFC. The findings suggested that Rg1 could exert antidepressant effects via HPA and HPG axes (Mou et al., 2017). In addition, in CUMS model, Rg1 significantly reduced BDNF expression and reduced PKA and CREB phosphorylation in the amygdala (Liu et al., 2016). CUMS induced miR-134 overexpression in vmPFC and the decrease of LIM-domain kinase 1 (Limk1) and cofilin. However, following Rg1 treatment, the above outcomes were reversed (Fan et al., 2018b).

In CMS mouse model of depression, Rg1 revealed antidepressant-like activity in FST and TST. The mechanism of action is to up-regulate BDNF signaling pathway in the hippocampus and down-regulate the level of serum corticosterone. Additionally, Rg1 can reverse the reduction in dendritic spine density and hippocampal neurogenesis caused by CMS (Jiang et al., 2012).

The astrocytes in the prefrontal cortex and hippocampus of main rats were treated with CORT and cultured for 24 h to induce gap junction damage. Rg1 pretreatment extensively improved Cx43 gap junctions of CORT-treated astrocytes, reversed the CORT-induced down-regulation of Cx43 biosynthesis, accelerated Cx43 degradation, and enhanced the two primary astrocytes cell Cx43 degradation pathway (Xia et al., 2020; Lenfant et al., 2021). Depression can be efficiently treated by raising Cx43 content, which may be owing to the effect of Rg1 on the degradation of ubiquitin-proteasome and autophagy-lysosome Cx43 pathways (Wang H. Q. et al., 2021). CORT considerably reduced astrocytes survival rate (Lou et al., 2020) affects gap junction function. Rg1 can alleviate CORT-induced gap junction dysfunction and may have clinical implications for treating depression (Xia et al., 2017). However, the effect of Rg1 (Lou et al., 2020)can be reversed by injecting the gap junction blocker carbenoxolone (CBX) into the CA1 area of the hippocampus on both sides. Moreover, Rg1 significantly improved antidepressant susceptibility caused by injecting phenoxyketone or Gap26 (a selective inhibitor of Cx43) into the animal’s PFC (Xia et al., 2020).

In CUS model, Rg1 can particularly improve the shortening of the diffusion distance of rat dye and expand the ultrastructure of abnormal astrocyte gap junctions in PFC. With the decrease of CUS exposure, Cx43 expression in PFC was increased (Jin et al., 2017; Lou et al., 2020), and the reduction of GFAP ratio also improved (Lou et al., 2020). In CUS model, the antidepressant effect of Rg1 may be mediated in part by defending the gap junctions of astrocytes in PFC (Jin et al., 2017).

The antidepressant-like effect of Rg1 was mediated in part by activating cAMP response element-binding protein BDNF system (Zhu et al., 2016)in PFC.

#### 3.2.8 Ginsenoside Rg2

In CMS mouse model, BDNF signaling pathway in the hippocampus was boosted due to Rg2 administration, which inhibited the depression-like effects induced by CMS, and knocking out TrkB can entirely block the antidepressant effect of Rg2 in mice (Ren et al., 2017).

#### 3.2.9 Ginsenoside Rg3

Literature researches have demonstrates that Rg3 has evident antidepressant effects (Zhang et al., 2017). In TST and FST, LPS-induced weight loss, anorexia, and immobility time can be successfully reversed by Rg3 pretreatment. The disordered turnover of tryptophan and serotonin in the hippocampus is impaired by Rg3, accompanied by reduced mRNA expression of pro-inflammatory cytokines and indoleamine-2,3-dioxygenase (IDO). This section is linked to microglia activation and nuclear factor kappa B (NF-κB) pathway regulation. Additionally, it can be seen that the levels of interleukin-6 (IL-6) and tumor necrosis factor-α (TNF-α) in plasma induced by LPS have a substantial decrease, and the metabolism of tryptophan-kynurenine is reestablished. Balance is necessary throughout the body. It can be seen that Rg3 effectively improves the depression-like behavior induced by immune activation (Kang et al., 2017). In HT22 mouse hippocampal neuronal cells treated with N-methyl-d-aspartic acid (NMDA), Rg3 alters the cell cycle to restore proliferation and avoid cell apoptosis. In FST, TST, and SPT, it is found that Rg3 can expressively reduce the expression levels of phosphorylated CREB and BDNF induced by CMS (Zhang et al., 2017).

Rg3 can effectively block the depression-like symptoms induced by CSDS and ultimately restore the reduction of the hippocampal BDNF signal pathway caused by CSDS. If a BDNF signal blocker is applied, it can block the antidepressant effect of Rg3 (You et al., 2017).

Furthermore, Rg3 can raise the level of NA in the brain of mice exposed to FST and act as an antidepressant without influencing 5-HT and DA (Zhang et al., 2016).

#### 3.2.10 Ginsenoside Rg5

Ginsenoside Rg5 is a low-toxic bioactive component of ginseng that has pharmacological effects on the central nervous system. Rg5 has antidepressant efficacy in FST and TST but does not affect locomotor activity. In CSDS model, Rg5 is also effective. It demonstrates the antidepressant activity by activating the hippocampal BDNF system that was reduced by CSDS influence. However, tyrosine kinase B (TrkB) inhibitors can make Rg5 lose its antidepressant effect, although tryptophan hydroxylase inhibitors will not (Xu et al., 2017).

#### 3.2.11 Ginsenoside Rh2

Ginsenoside Rh2 has a therapeutic effect on various disorders (Lv et al., 2021). Experiments revealed that CRC mice treated with Rh2 had improved depression-like behaviors in all FST, TST, and SIT. It appears to be accomplished by altering the expression of depression-related factors, including IL-6, IL-18, and TNF-α to achieve antidepressant-like effects. Furthermore, experiments show that, compared to the control group, CRC mice treated with Rh2 have a longer life duration. It can be demonstrated that Rh2 can successfully alleviate tumor-related depression in CRC mice, which could pave the way for a new strategy of treating CRC-related depression (Wang J. et al., 2016).

#### 3.2.12 Ginsenoside Rk1

Ginsenoside Rk1 is a saponin formed by thermal processing that exhibits anti-inflammatory and anti-tumor effects. Through LPS model, it can be seen that Rk1 enhances the activity of antioxidant enzyme SOD in the brain and inhibits lipid peroxidation. Different Rk1 concentrations can inhibit the expression of TNF-α and IL-1 in serum to changing degrees and reduce the concentration of IL-6. Western blotting revealed that Rk1 considerably down-regulated the expression of Sirt1 and inhibited the ratio of p-NF-κb/NF-κb and p-IκB-α/IκB-α, protecting nerves and resisting depression-like behaviors. Besides, under the action of Rk1, the expression levels of BDNF and TrkB induced by LPS decreased significantly. It illustrates that Rk1 can effectively inhibit neuroinflammation and positively regulate BDNF-TrkB pathway through its antioxidant activity (Li Z. et al., 2020).

### 3.3 Other Derived Compounds From *Panax notoginseng*

#### 3.3.1 Ginsenoside 20(S)-Protopanaxadiol

Ginsenoside 20(S)-protopanaxadiol (20(S)-PPD) is an aglycone derivative of the Rb1 metabolite. In CUMS model, Ginsenoside 20(S)-protopanaxadiol efficiently controls serum CORT and pro-inflammatory cytokines (IL-6, IL-1β, and TNF-α) and neurotransmitters (5-HT and NE) induced by CUMS in the hippocampus and PFC, inhibit microglia activation in DG induced by CUMS. The consequences revealed that PPD down-regulated the levels of iNOS, COX2, cleaved-caspase3, cleaved-caspase9, Bax, Bcl-2, and ac-p65 in rat’s hippocampus up-regulated Sirt1 levels. It has a positive effect on CUMS model. Depression-like effects are partially achieved by modifying the HPA axis’ malfunction, reestablishing neurotransmitter levels, and preventing neuronal death and neuroinflammation through SIRT1/NF-kB signaling pathway (Jiang et al., 2020a).

Through oral administration of PPD to rats, it was found in FST, TST, and rat olfactory bulb resection depression models that PPD has the same antidepressant activity as fluoxetine, but whether its antidepressant mechanism is consistent with the existing antidepressant mechanism The difference is currently unclear. Because PPD is not like fluoxetine, it does not reduce the oxidative stress and serum CORT concentration in the brain of olfactory bulb resection rats to achieve antidepressant-like behavior. It has not been demonstrated that PPD treatment interferes with the normal function of CNS in rats with olfactory bulb resection.

Additionally, monoamine neurotransmitter levels in the brains of rats treated with PPD olfactory bulb resection are higher, and the *in vitro* reuptake test also demonstrates that PPD has a modest inhibitory effect. Because the monoamine reuptake activity of PPD is relatively low, it is unknown whether its antidepressant mechanism differs from currently available antidepressants (Xu et al., 2010).

#### 3.3.2 Ginsenoside Metabolite Compound K (CK)

Ginsenoside metabolite compound K [C-K; 20-O-(beta-d-glucopyranosyl)-20(S)-protopanaxadiol] has anti-inflammatory and significant pharmacological effects on CNS (Song et al., 2018). The antidepressant efficacy of CK is similar to that of Rb3 (Zhang et al., 2016). FST, TST, and sports activities were performed in a behavioral despair mice model, open field tests, food consumption, and SPT were examined in a CUMS rat model. The results revealed that CK elevated the immobility time of mice in FST and TST, which D1 receptor antagonist Sch23390 can partially reverse. In CUMS model, CK improved the depression-like behavior of rats, increased 5-HT and DA concentrations, and their metabolites in PFC and hippocampus of rats, and reversed the overexpression of MAO B in PFC and hippocampus. GSH and GPx in PFC and hippocampus have also been improved. Additionally, immunohistochemistry (IHC) results demonstrated that CK up-regulated BDNF and NGF in rats. In summary, the antidepressant effects of CK may be attributed to its ability to modulate the concentration of monoamine neurotransmitters and its antioxidant capacity and neurotrophic factor levels in CNS.

Furthermore, NA concentration in the brain of FST mice was considerably increased by the influence of CK (Zhang et al., 2016). In contrast, the concentration of 5-HT and DA were unaffected (Zhang et al., 2016).

#### 3.3.3 Ginseng Extract G115^®^

Co-administration of G115^®^ can boost the effect of fluoxetine. Autopsy tissue analysis revealed no change in Trkb expression but a substantial difference in the expression of BDNF concentration in the left hippocampus and the left prefrontal cortex (Terstege et al., 2021).

#### 3.3.4 Ginsenoside H Dripping Pills (GH)

Ginsenoside H Dropping Pill (GH) is an auxiliary medicine for cancer treatment. Its primary constituent is Rh2. Early investigations have revealed that Rh2 can dramatically inhibit the growth of U14 cervical cancer tumor-bearing mice. Additionally, Rh2 has been demonstrated to alleviate depressive behavior in mice. In other words, GH inhibits tumor growth and acts as an antidepressant. It gives an exclusive drug for treating depression in cancer patients and has research value.

In CUMS model, GH has been successfully shown to have an antidepressant effect. The intermediate-dose GH (56 mg/kg) has the best inhibitory effect on depression-like behavior induced by CUMS in rats. After network pharmacology analysis, GH may exert antidepressant-like effects by regulating cAMP signaling pathway. The chief target proteins cAMP, PKA, CREB, p-CREB, and BDNF were verified in CUMS model rats in cAMP signaling pathway. It was found that GH can activate cAMP-PKA-CREB-BDNF signaling pathway (Zhao et al., 2020).

## 4 Discussion and Conclusion

*Panax notoginseng* is traditional Chinese herbal medicine with valuable efficacy and less toxicity. According to recent research, *Panax notoginseng* discovered that its active constituents could treat depression. The compounds of *Panax notoginseng* effectively treated depression through modulating neurotransmitters (5-HT and DA), brain-derived neurotrophic factor (BDNF), and its intracellular signaling pathway, protecting neurons by anti-inflammatory anti-oxidation, and regulation of protein expression related to depression. Indeed, serotonergic, dopaminergic, and noradrenergic systems interact with *Panax notoginseng*’s antidepressant properties. Increasing monoamine synaptic availability is an essential component of ginsenoside regulation mechanism.

Meanwhile, ginsenosides’ “melioration effect” is linked to normalization, reestablishing neural plasticity, and alleviating stress-induced nerve injury. Other negative stimuli are more likely to contribute to the recovery from depression as disrupted neuroplasticity is a vital feature of brain in response to depression. Since some specific brain regions are involved in the pathogenesis of depression. The antidepressant-like effects of *Panax notogensing* ingredients affected specific brain regions. Most compounds such as Rb1, Rd, Re, Rg5, Rk1 mainly function in hippocampus. SLPN, Rb3, Rf, Rg3, PDD, CK, G115^®^, etc. affect the prefrontal cortex region while Rg1 act on the amygdala part and Rf influence the olfactory bulb. Moreover, ginsenosides Re also have an effect on the locus coeruleus of the brainstem. Furthermore, research also found that Rb1 could inhibit the activity of L-type voltage-gated calcium channels and Rf may inhibit inward currents in oocytes of sensory inhibitory sensory neuron N-type, other calcium channels, and nicotinic acetylcholine receptor subtypes. However, the specific neuronal cells or circuits that may targeted by Panax notoginseng were not fully understood and need further investigation.

Studies has revealed that active ingredient Rg1 does not exert its antidepressant effect through the monoaminergic system, and its mechanism is different from conventional antidepressants (Jiang et al., 2012). The dose of Rg1 used to treat depression may be ineffective in saving PTSD-like behavior in a mice model of single-prolonged stress (Sun et al., 2020). Although the main chemical composition of *Panax notoginseng* is identical to that of ginseng, the unique ingredients of *Panax notoginseng* and the ginsenosides in it, our research on their mixtures or monomers is not yet fully detailed (Xie et al., 2018).

Some ingredients in *Panax notoginseng* have definite effects on depression, but they have different effects on various animal models. Therefore, we have summarized the therapeutic effects of most ingredients in *Panax notoginseng,* which may provide new insight into potential healing targets and pharmacological properties of *Panax notoginseng* and its main active ingredients. However, additional pre-clinical and clinical research is required to elucidate the detailed antidepressant mechanisms of *Panax notoginseng*. Besides, there are lack of studies that focus on long-lasting anti-depressive effects of *Panax notoginseng* suggested that we should pay more attention to the long-lasting effects in the future research. Apart from chemical compounds, the advancement of technology, optogenetics has been used more and more widely in the field of depression (Chen et al., 2022). One study showed that optogenetic stimulation of mice produced rapid and stimulating antidepressant effects (Hare et al., 2019). Until now, there are less research has investigated whether the combination of optogenetics and chemical drugs can produce a synergistic antidepressant effect. Therefore, chemical drugs especially the compounds from Panax notoginseng with anti-depressive effects combined with optogenetic stimulation may provide a promising therapy for treating depression.

## Glossary

DOPAC: 3,4-Dihydroxyphenylacetic acid
(5-HT): 5-hydroxytryptamine: Serotonin
(ac-p65): Acetylated p65
(ACTH): Adrenocorticotropic hormone
(AR): Androgen receptor
(B + GRb1): Berberine treatment with ginsenoside Rb1
(BDNF): Brain-derived neurotrophic factor
(CBX): carbenoxolone
(CHM): Chinese herbal medicine
(CCI): Chronic contractile injury
(CRS): Chronic restraint stress
(CSDS): Chronic social failure stress Model
(CUMS): Chronic unpredictable mild stress
(CK): Compound K
(CORT): Corticosterone
(EC): Escherichia coli
Gap26: (a selective inhibitor of Cx43)
(20(S)-PPD): Ginsenoside 20(S)-protopanaxadiol
(GH): Ginsenoside H Dropping Pill
(Rb1): Ginsenoside Rb1
(GFAP): Glial fibrillary acidic protein
(GR): Glucocorticoid receptor
(HVA): homovanillic acid
(IS): Immobilization stress
(IHC): Immunohistochemistry
(IDO): Indoleamine-2,3-dioxygenase
(IL18)
Interleukin-18
(IL-6): Interleukin-6
L-AAA: (La-amnoadepate)
(Limk1): LIM-domain kinase 1
(LPS)Lipopolysaccharide(LC): Lipopolysaccharide(LC)Locus coeruleus
(MAOI): Monoamine oxidase inhibitors
(NMDA): N-methyl-d-aspartic acid
(NE): Norepinephrine
(NF-κB): Nuclear factor kappa B
OGD: (oxygen glucose deprivation)
(PNS): Panax notoginseng saponins
(PFC): prefrontal cortex
(PRS): Prenatally stressed dams
(5-HT): 5-hydroxytryptamine: Serotonin
(SSRIs): Serotonin reuptake inhibitors
SOD: (Superoxide dismutase)
(SLPN): Total saponins from the caudexes and leaves of Panax notoginseng
(TCA): Tricyclic antidepressants
(TNF-α): Tumor necrosis factor-α
(TH): Tyrosine hydroxylase
(TrkB): Tyrosine kinase B
(vmPFC): Ventral medial prefrontal cortex )
